# Folic acid supplementation during pregnancy and postpartum depressive symptoms

**DOI:** 10.11606/s1518-8787.2023057004962

**Published:** 2023-10-24

**Authors:** Bárbara Heather Lutz, Iná da Silva dos Santos, Marlos Rodrigues Domingues, Joseph Murray, Mariângela Freitas da Silveira, Vanessa Irribarem Avena Miranda, Marysabel Pinto Telis Silveira, Sotero Serrate Mengue, Tatiane da Silva dal Pizzol, Andréa Dâmaso Bertoldi

**Affiliations:** I Universidade Federal de Pelotas Departamento de Medicina Social Programa de Pós-Graduação em Epidemiologia Pelotas RS Brasil Universidade Federal de Pelotas . Departamento de Medicina Social . Programa de Pós-Graduação em Epidemiologia . Pelotas , RS , Brasil; II Universidade Federal de Pelotas Programa de Pós-Graduação em Educação Física Pelotas RS Brasil Universidade Federal de Pelotas . Programa de Pós-Graduação em Educação Física . Pelotas , RS , Brasil; III Universidade do Extremo Sul Catarinense Programa de Pós-graduação em Saúde Coletiva Criciúma SC Brasil Universidade do Extremo Sul Catarinense . Programa de Pós-graduação em Saúde Coletiva . Criciúma , SC , Brasil .; IV Universidade Federal de Pelotas Departamento de Fisiologia e Farmacologia Programa de Pós-Graduação Multicêntrico em Ciências Fisiológicas Pelotas RS Brasil Universidade Federal de Pelotas . Departamento de Fisiologia e Farmacologia . Programa de Pós-Graduação Multicêntrico em Ciências Fisiológicas . Pelotas , RS , Brasil; V Universidade Federal do Rio Grande do Sul Faculdade de Medicina Programa de Pós-Graduação em Epidemiologia Porto Alegr RS Brasil Universidade Federal do Rio Grande do Sul . Faculdade de Medicina . Programa de Pós-Graduação em Epidemiologia . Porto Alegr e, RS , Brasil

**Keywords:** Pregnancy, Folic Acid, Depression, Postpartum, Cohort Studies

## Abstract

**OBJECTIVE:**

To verify whether folic acid supplementation during pregnancy is associated with the occurrence of maternal depressive symptoms at three months postpartum, in the 2015 Pelotas Birth Cohort.

**METHODS:**

This study included 4,046 women, who were classified into three groups: did not use folic acid supplementation during pregnancy; used during only one trimester of pregnancy; and used for two or three trimesters. Depressive symptoms were assessed at three months postpartum using the Edinburgh Postnatal Depression Scale (EPDS), at cutoff points ≥ 10 (mild symptoms) and ≥ 13 (moderate to severe intensity).

**RESULTS:**

The overall prevalence of mild symptoms was of 20.2% (95%CI 19.0–21.5), and moderate and severe was 11% (95%CI 10.0–12.0). The prevalence of EPDS ≥ 10 was of 26.8% (95%CI 24.0–29.5) among women who did not use folic acid and 18.1% for both those who used it during one trimester of pregnancy (95%CI 16.1–20.1) and those who used it for two or three trimesters (95%CI 16.0–20.2). The prevalence of EPDS ≥ 13 was of 15.7% (95%CI 13.5–17.9) in those who did not use folic acid, 9.1% (95%CI 7.5–10.6) in those who used it for one trimester, and 9.4% (95%CI 7.8–11.0) in those who used it for two or three trimesters. In the adjusted analyses, there was no statistically significant association between the use of folic acid during pregnancy and the occurrence of depressive symptoms at three months postpartum.

**CONCLUSION:**

There was no association between folic acid supplementation during pregnancy and postpartum depression at three months.

## INTRODUCTION

Depressive disorders are classified by the World Health Organization (WHO) as the largest contributor to global disability, particularly among women ^1 , 2^ . They are characterized by sadness, loss of interest or pleasure, feelings of guilt or low self-worth, disturbed sleep or appetite, feelings of tiredness, and poor concentration, which can be long-lasting or recurrent, substantially impairing the individual’s ability to cope with daily life ^1 , 2^ .

Postpartum depression (PPD) is a common complication of pregnancy ^3^ , in which the severity depends on factors such as socioeconomic context and family support ^4^ . PPD has adverse consequences not only for the mother, but also for the family since it can affect child development (for example, it is associated with a higher risk of low birth weight in the first year of life ^5^ ), create difficulties in establishing mother-fetal bonding ^6^ , and cause impairment in social, affective, and cognitive development aspects ^7 , 8^ .

A systematic review of 16 international studies, including approximately 35,000 women, indicated a mean prevalence of 17% for puerperal women with a high probability of having PPD. Most studies used the Edinburgh Postnatal Depression Scale (EPDS), with cutoff points ranging from 9 to 13 ^9^ . Data from a Brazilian study with approximately 23,000 women from all regions of the country indicated an even higher prevalence, of approximately 25%, using EPDS ≥ 13 ^10^ .

The etiology of PPD is believed to be linked to biological, genetic, hormonal, psychosocial and environmental factors ^3^ . Deficiency of nutritional factors such as folate/folic acid, vitamin B12, polyunsaturated fatty acids, selenium, zinc, calcium, and iron has also been associated with PPD ^3 , 11^ . Folic acid is part of the vitamin B complex (vitamin B9). A meta-analysis of observational studies showed that low folate levels are associated with depression in the general population ^14^ . Folate is a major determinant of 1-carbon metabolism, where S-Adenosylmethionine (SAM) is formed. SAM donates important methyl groups for neurological function. Furthermore, increased plasma homocysteine is a functional marker of folate deficiency, and elevated homocysteine levels are found in depressive patients ^15 , 16^ .

WHO recommends daily oral supplementation of iron and folic acid as part of prenatal care to reduce the risk of low birth weight, maternal anemia, and iron deficiency ^17^ . In Brazil, folic acid supplementation is indicated at least 30 days before conception until the end of pregnancy, at a dose of 0.4mg per day, to prevent neural tube defects and anemia ^18^ . There are few published studies that evaluated the association between folic acid supplementation or folate levels during pregnancy and the occurrence of PPD ^3 , 19^ , only two of which were population-based ^20 , 21^ . Thus, the objective of this study is to evaluate the prevalence of depressive symptoms at three months after childbirth and its relationship with the use of folic acid supplementation among mothers belonging to the 2015 Pelotas Birth Cohort ( *Coorte de Nascimentos de Pelotas de* 2015 – C2015).

## METHODS

The data from this study are part of C2015, held in the city of Pelotas, in the state of Rio Grande do Sul, in southern Brazil. All women who gave birth in the five maternity hospitals in Pelotas, from January 1, 2015, to December 31, 2015, and who lived in the urban area of the municipality and in Colônia Z3, as well as in the Jardim América neighborhood, adjacent to Pelotas and belonging to the municipality of Capão do Leão, were invited to participate in the study. Methodological details can be found elsewhere ^23^ .

This cohort started in 2014, in the prenatal period. The 123 health units and private clinics that offer prenatal care in the city were visited weekly, between May 2014 and December 2015, to identify pregnant women with a probable date of delivery for the year 2015. These women were visited at home or invited to attend the research clinic, between 16 and 24 weeks of gestation, to answer a health questionnaire, including questions about the use of medications and vitamins ^23^ .

In the perinatal study, mothers were interviewed after delivery, during their stay in the maternity ward, answering a standardized questionnaire about the prenatal period, including the use of medications and vitamins. Given that 75% of the puerperal women had also been interviewed during the C2015 prenatal study, it was possible to complement the information on the use of medications and vitamins captured in the perinatal study with data obtained at a time closer to their use. This strategy allowed qualifying the information, bearing in mind that, during the prenatal period, the recall period was shorter, and, as the interviews were carried out at home, it was possible to verify the prescriptions and packaging of the products used.

In both the prenatal and perinatal studies, information regarding the use of folic acid was obtained through the following questions: “Have you used or are you using any vitamins, calcium, folic acid or iron salts since you became pregnant?” If so, the names of these drugs were questioned and, later, for each drug reported, the following questions were asked, with the aim of characterizing its use: “In which trimester of pregnancy did you use this drug?” 1st trimester (up to week 13), 2nd trimester (week 14 to 27), and 3rd trimester (week 28 onwards or still in use). For the current study, we considered the use of folic acid alone or in association with other vitamins and/or mineral salts, regardless of dosage.

The three-month postpartum follow-up was performed at the research clinic. On that occasion, maternal depressive symptoms were assessed using the EPDS. This scale consists of 10 items, each with a score ranging from zero to three. The EPDS was validated for use in Brazil, with the cutoff point ≥ 10 being considered the best to identify mothers with mild depressive symptoms in the postpartum period, with a sensitivity of 82.7% (75.3–89.9) and specificity of 65.4% (59.8–71.1) ^24^ ; and the cutoff ≥ 13 for depressive symptoms of moderate to severe intensity, with sensitivity of 59.6% (49.5–69.1) and specificity of 88.3% (83.9–91.9) ^24^ . For this study, mothers who attended the three-month follow-up but did not respond to the EPDS were excluded.

The potential confounders analyzed were: age (< 20, 20–34, and ≥ 35 years); color (white, black, and mixed/other); education (0–4, 5–8, 9–11, and ≥ 12 years of schooling); income in minimum wages (MW), considering the value of R$788.00 in force during the year 2015 ( ≤ 1, 1.1–3, 3.1–6 , 6.1–10, and > 10); parity (1, 2, 3, and ≥ 4 children); living with a partner (yes/no); support from the baby’s father during pregnancy (a lot, more or less/a little, no support); depressive symptoms during pregnancy (EPDS ≥ 11) ^25^ , reported in the prenatal study; trimester of prenatal care initiation (1st, 2nd, or 3rd); number of prenatal consultations (< 6 or ≥ 6); smoking during pregnancy (yes/no); drinking during pregnancy (yes/no); and physical activity during pregnancy (150 minutes or more per week in at least one trimester) (yes/no). Age was collected in complete years and subsequently categorized. Skin color was self-reported by the mothers. Education was collected in complete years of schooling and subsequently categorized. Family income was reported in reais and later categorized into MW. For parity, the current pregnancy was also considered, without including stillbirths, and categorized later. For a complementary analysis, the use of antidepressant drugs from birth to three months postpartum (yes/no) was considered.

Data analysis was performed using the Stata ^®^ statistical program, version 12.1. The sample was described showing the proportions of independent variables and 95% confidence intervals (95%CI). Women were classified into three groups regarding exposure to folic acid: did not use of folic acid during pregnancy, used during only one trimester of pregnancy, and used folic acid during two or three trimesters. The mean and standard deviation (SD) of the continuous EPDS score and the prevalence of the EPDS ≥ 10 and EPDS ≥ 13 outcomes, with 95%CI, were calculated for each category of folic acid use.

Adjusted analyses were performed using Poisson regression for the two outcomes: EPDS ≥ 10 and EPDS ≥ 13. Variables were selected in backward mode, with p-values < 0.20 maintained in the model. The significance level adopted to consider statistically significant associations was 0.05. The variables age, education (years of schooling), income, number of prenatal consultations and parity were analyzed continuously. All variables included in the model were tested for their association with the use of folic acid and postpartum depressive symptoms, showing statistically significant relationships.

Additionally, two complementary analyses were performed: A) by multinomial logistic regression, with the outcome in three categories: EPDS < 10, EPDS 10 to 12, and EPDS ≥ 13; and B) by linear regression, with the EPDS outcome in continuous form. An interaction test was also performed between the use of folic acid and depressive symptoms during pregnancy (EPDS ≥ 11).

The study was approved by the Ethics Committee of the *Escola Superior de Educação Física, Universidade Federal de Pelotas* , under Protocol 522,064, registered on Plataforma Brasil. All interviews were carried out after signing the informed consent form by the mothers.

## RESULTS

The 2015 Pelotas Birth Cohort is composed of live births of 4,220 women. There were 160 losses at the three-month follow-up, and 14 women did not answer the EPDS. The EPDS score at three months postpartum ranged from 0 to 27, with a mean of 5.99 points (SD = 4.83). The prevalence of mild depressive symptoms (EPDS ≥ 10) was 20.2% (95%CI: 19.0–21.5), and of moderate and severe symptoms (EPDS ≥ 13), 11.0% (95%CI: 10.0–12.0).

Table 1 presents the characteristics of the analyzed sample, according to the independent variables and the comparison with the original cohort (initial sample collected in the perinatal study). Most mothers reported being white (70.8%), aged between 20 and 34 years (70.8%), living with a partner (85.8%), having received a lot of support from the baby’s father during pregnancy (89 %), having started prenatal care in the first trimester of pregnancy (55.5%), and having had six or more consultations (86.6%). Just over a third (34.5%) had 9 to 11 years of schooling, about half (47.2%) had a family income of 1.1 to 3 MW, and 50.0% were primiparous. Regarding health behaviors, 7.4% reported having consumed alcohol during the gestational period, 16.1% were smokers, and only 10.1% practiced physical activity during this period. Among the women who had been enrolled in the prenatal study (n = 3,029), 24% had an EPDS ≥ 11. There were no statistically significant differences between the original cohort and the analyzed cohort regarding the independent variables ( Table 1 ).

**Table 1:** Comparison between the original cohort and study participants. Pelotas Birth Cohort, 2015.

Maternal characteristics	Original cohort (n = 4,220)		Participants* (n = 4,046)	
	
	n	% (95%CI)	n	% (95%CI)
Age (years)				
< 20	619	14.7 (13.6–15.7)	593	14.7 (13.6–15.8)
20–34	2,981	70.7 (69.3–72.0)	2,864	70.8 (69.4–72.2)
≥ 35	619	14.7 (13.6–15.7)	588	14.5 (13.4–15.6)
Family income (minimum wages)				
≤ 1	534	12.7 (11.7–13.7)	506	12.5 (11.5–13.5)
1.1–3.0	1,991	47.2 (45.7–48.7)	1,910	47.2 (45.7–48.8)
3.1–6.0	1,115	26.4 (25.1–27.8)	1,077	26.6 (25.3–28.0)
6.1–10.0	316	7.5 (6.7–8.3)	299	7.4 (6.6–8.2)
> 10.0	262	6.2 (5.5–6.9)	252	6.2 (5.5–7.0)
Education (years of schooling)				
0–4	387	9.2 (8.3–10.0)	361	8.9 (8.0–9.8)
5–8	1,084	25.7 (24.4–27.0)	1,041	25.7 (24.2–27.1)
9–11	1,442	34.2 (32.7–35.6)	1,397	34.5 (33.1–36.0)
≥ 12	1,306	31.0 (29.6–32.4)	1,246	30.8 (29.4–32.2)
Color				
White	2,982	70.8 (69.4–72.2)	2,861	70.8 (69.4–72.2)
Black	661	15.7 (14.6–16.8)	635	15.7 (14.6–16.8)
Mixed/other	570	13.5 (12.5–14.6)	543	13.4 (12.4–14.5)
Lives with partner				
Yes	3,620	85.8 (84.7–86.9)	3,471	85.8 (84.7–86.9)
No	599	14.2 (13.1–15.3)	574	14.2 (13.1–15.3)
Baby’s father support				
A lot of support	3,690	89.0 (88.1–90.0)	354	89.0 (88.0–90.0)
More or less/little support	297	7.2 (6.4–8.0)	288	7.2 (6.4–8.0)
No support	157	3.8 (3.2–4.4)	150	3.8 (3.2–4.4)
Parity (live births only)				
1	2,108	50.0 (48.5–51.5)	2,023	50.0 (48.5–51.6)
2	1,306	31.0 (29.6–32.4)	1,260	31.1 (29.7–32.6)
3	4,461	10.9 (10.0–11.9)	443	11.0 (10.0–11.9)
≥ 4	343	8.1 (7.3–9.0)	319	7.9 (7.1–8.7)
Number of prenatal consultations				
< 6	577	14.0 (13.0–15.1)	531	13.4 (12.3–14.5)
≥ 6	3,538	86.0 (84.9–87.0)	3,429	86.6 (85.5–87.7)
Trimester of prenatal care initiation				
First	2,058	54.9 (53.3–56.5)	2,013	55.5 (53.8–57.1)
Second	1,461	39.0 (37.4–40.6)	1,396	38.5 (36.9–40.0)
Third	228	6.1 (5.3–6.9)	221	6.1 (5.3–6.9)
Depressive symptoms in pregnancy**				
Yes	746	24.2 (22.6–25.7)	726	24.0 (22.4–25.5)
No	2,342	75.8 (74.3–77.4)	2,303	76.0 (74.5–77.6)
Drinking during pregnancy				
Yes	314	7.4 (6.7–8.2)	301	7.4 (6.6–8.3)
No	3,903	92.6 (91.8–93.3)	3,743	92.6 (91.7–93.4)
Smoking during pregnancy				
Yes	698	16.6 (15.4–17.7)	653	16.1 (15.0–17.3)
No	3,519	83.4 (82.3–84.6)	3,391	83.9 (8.27–85.0)
Physical activity during pregnancy (≥ 150 min/week in at least one trimester)				
Yes	423	10.0 (9.1–10.9)	407	10.1 (9.1–11.0)
No	3,797	90.0 (89.1–90.9)	3,639	89.9 (89.0–90.9)
Use of folic acid during pregnancy				
Yes	3,080	74.1 (72.8–75.4)	2,966	74.4 (73.0–75.7)
No	1,076	25.9 (24.6–27.2)	1,021	25.6 (24.3–27.0)

95%CI: 95% confidence interval.

*Participants in the 3-month follow-up with information on postpartum depressive symptoms.

**n = 3,088 women evaluated in the prenatal study using the Edinburgh scale, considering the cutoff point ≥ 11 ^25^ .

Among the 4,046 women included in the current analysis, 3,987 reported information about folic acid use. Among these, 2,966 (74.4%; 95%CI: 73.0–75.7) reported having used folic acid during pregnancy. Among those who used it, 88.4% (n = 2,621) used an exclusive folic acid supplement (without any other vitamins or mineral salts) at some point during pregnancy. The Figure shows the trimesters of folic acid use by women participating in the study. Of the 2,966 women who reported having used folic acid during pregnancy, only 2,689 informed the trimester of use. Among these, 43.8% used folic acid only during the first trimester.

**Figure 1:**
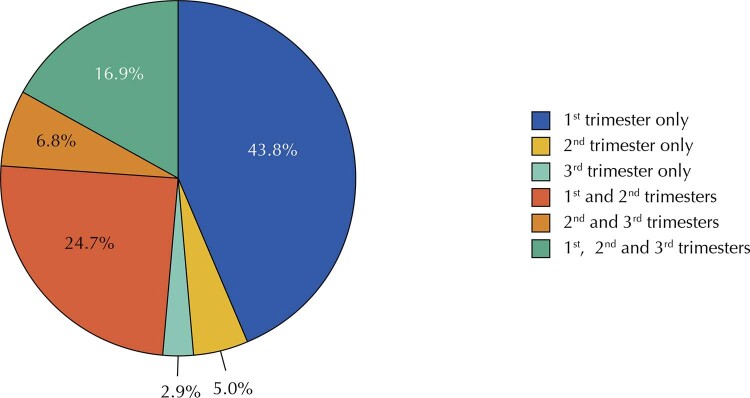
Trimesters of folic acid use during pregnancy (n = 2,689). Pelotas Birth Cohort, 2015.

Table 2 shows the mean and SD of EPDS at three months and the prevalence of outcomes EPDS ≥ 10 and ≥ 13 according to exposure categories. Among women who did not use folic acid during pregnancy, the mean EPDS score was 6.8 (SD = 5.3); among those who used it during one trimester, 5.7 (SD = 4.7); and among those who used it for two or three trimesters, 5.7 (SD = 4.6).

**Table 2:** Mean and standard deviation of the continuous score and prevalence of outcomes in each exposure category. Pelotas Birth Cohort, 2015.

Exposure	Mean and SD of EPDS score	EPDS ≥ 10	EPDS ≥ 13
	
		(95%CI)	(95%CI)
No use of folic acid (n = 1,020)	6.8 (5.3)	26.8 (24.0–29.5)	15.7 (13.5–17.9)
Use in 1 trimester (n = 1,391)	5.7 (4.7)	18.1 (16.1–20.1)	9.1 (7.5–10.6)
Use in 2 or 3 trimesters (n = 1,298)	5.7 (4.6)	18.1 (16.0–20.2)	9.4 (7.8–11.0)

SD: standard deviation; EPDS: Edinburgh Postnatal Depression Scale; 95%CI: 95% confidence interval.

Among women who did not use folic acid, the prevalence of EPDS ≥ 10 was 26.8% (95%CI: 24.0–29.5), and 18.1% both among those who used it during one trimester of pregnancy (95%CI: 16.1–20.1) and among those who used it for two or three trimesters (95%CI: 16.0–20.2) ( Table 2 ). The prevalence of EPDS ≥ 13 was 15.7% (95%CI: 13.5–17.9) among women who did not use folic acid, 9.1% (95%CI: 7.5–10.6) among those who used it for one trimester and 9.4% (95%CI: 7.8–11.0) among those who used it for two or three trimesters ( Table 2 ).

Table 3 shows the prevalence ratios for EPDS ≥ 10 and EPDS ≥ 13, with the respective 95%CI, obtained in the raw analysis and in the adjusted models. For mild depressive symptoms (EPDS ≥ 10), the variables that remained for adjustment in the final model were schooling, support from the baby’s father, drinking during pregnancy, parity, and depressive symptoms during pregnancy. As for moderate and severe symptoms (EPDS ≥ 13), the variables that remained in the final model were “living with a partner”, “parity,” and “depressive symptoms during pregnancy”.

**Table 3:** Associations between folic acid use during pregnancy and postpartum depressive symptoms. Pelotas Birth Cohort, 2015.

Variables	Raw analysis (n = 3,709)		Adjusted analysis (n = 2,810)*	
	
	PR (95%CI)	p-value	PR (95%CI)	p-value
EPDS ≥ 10				
No use of folic acid	1	< 0.001	1	0.112
Use for a single trimester	0.68 (0.58–0.79)		0.96 (0.80–1.14)	
Use for two or three trimesters	0.68 (0.58–0.79)		0.87 (0.72–1.04)	
EPDS ≥ 13				
No use of folic acid	1	< 0.001	1	0.107
Use for a single trimester	0.58 (0.46–0.72)		0.84 (0.65–1.10)	
Use for two or three trimesters	0.60 (0.48–0.75)		0.80 (0.61–1.04)	

95%CI: 95% confidence interval; EPDS: Edinburgh Postnatal Depression Scale.

*Adjusted analysis (Poisson Regression) for age, schooling, ethnicity, income, parity, living with a partner, support from the baby’s father, trimester of initiation of prenatal care, number of prenatal consultations, smoking during pregnancy, drinking during pregnancy, physical activity during pregnancy and depressive symptoms during pregnancy (EPDS ≥ 11).

In the raw analysis, the use of folic acid was significantly associated with a decrease in the risk of postpartum depressive symptoms at three months, both among users of folic acid for only one trimester and among those who used it for two trimesters or more, in both EPDS cutoff points ( Table 3 ). In the adjusted analyses, however, there was no statistically significant association between use of folic acid during pregnancy and occurrence of depressive symptoms at three months postpartum, considering the two cutoff points.

In the complementary analyses, both in the multinomial logistic regression and in the linear regression, the results were similar and in the same direction of the Poisson regression analyses. The interaction test did not indicate that the variable “depressive symptoms during pregnancy” modified the association between folic acid use and depressive symptoms in the postpartum period. Additionally, control was also performed for antidepressant use, with no changes in the results. Only 23 women (0.5% of the sample) were using antidepressants from birth to three months postpartum. Seven of them scored above 10 on the Edinburgh questionnaire, four of them with moderate to severe symptoms.

## DISCUSSION

The prevalence of mild depressive symptoms at three months postpartum was 20.2% and of moderate to severe depressive symptoms, 11%. The use of folic acid during pregnancy had a protective effect in the raw analysis for both cutoff points, but lost significance in the adjusted analyses.

Several studies have reported the relationship between folate and depression in the general population ^14 , 16 , 26 , 27^ , but there is not much evidence in relation to PPD. One study observed low folate and vitamin B12 serum levels and high homocysteine levels in women of childbearing age with psychotic disorders ^28^ . Folic acid supplements have already been studied as an adjuvant treatment for depression ^15 , 29^ , and folic acid rich diets have suggested a reduced risk of depression in some populations ^30 , 31^ . The study by Yan ^3^ found a lower risk for PPD in pregnant women who had taken folic acid supplementation for more than six months, compared with those who supplemented for less than six months. Possible hypotheses for this difference and limitations of that study would be the exclusion of women who did not use folic acid or who used it only in the preconception period, and the non-inclusion of depression symptoms during the gestational period as a confounding factor. The study was carried out with women who underwent postpartum review at maternal and child health centers in Tianjin, China, and used a scale designed for screening depressive symptoms in the general population ^3^ .

Other studies found no relationship between folic acid supplementation/folate levels and postpartum depression. Blunden et al. ^21^ found no significant differences between women with or without postpartum depressive symptoms in terms of folate concentration in red blood cells or dietary intake of folate, vitamin B12 and vitamin B6, before or during pregnancy. Chong et al. ^19^ also did not observe differences in plasma folate concentrations in women with and without PPD, but folate concentrations were significantly lower among those with probable gestational depression than among those without symptoms. Miyake et al. ^22^ found no association between intake of folate, cobalamin or pyridoxine and the risk of PPD. In the study by Lewis et al. ^20^ found no evidence that folic acid supplementation would reduce the risk of depression during pregnancy and up to eight months postpartum. However, the same study showed that folic acid supplements during pregnancy protected against depression 21 months postpartum, and that this effect was more pronounced in women with the MTHFR C677T TT genotype.

The plausibility for the potential protective effect of folate on the occurrence of PPD stems from the fact that this and other nutrients are important in the neurotransmission system, and pregnancy tends to lead to its depletion ^12 , 13 , 32^ . Folate concentrations in maternal serum and erythrocytes decrease from the fifth month of pregnancy and tend to remain low for a long period after delivery ^33^ . Folate, vitamin B12, and vitamin B6 are critical factors in the metabolism of homocysteine, which is a necessary precursor in the biosynthesis of the neurotransmitters serotonin, dopamine, and norepinephrine, which are implicated in the pathogenesis of depression ^27 , 32^ .

Two randomized trials, published in the early 1990s, demonstrated that folic acid supplementation prevented the occurrence and recurrence of neural tube defects ^34 , 35^ , thus recommending universal supplementation in the preconception period and during pregnancy to prevent these defects, as well as to prevent anemia ^17 , 18^ . During the study period, the current protocol in Brazil recommended the use of folic acid during the preconception period and only in the first gestational trimester ^4^ . However, it is common not to have this supplement prescribed when the woman begins prenatal care late ^36^ .

This study has some limitations. It was not possible to evaluate the dosage of the supplements used, only the information on use by gestational trimester was analyzed, but without the guarantee that the supplement was used during the entire period of the trimester in question. In addition, our analysis is based on self-report, with no dietary recall, consumption of folic acid fortified foods or serum folate levels. Likewise, it was not possible to evaluate the use of supplementation in the preconception period.

Among the strengths of this study is the fact that it is a population-based cohort, with a large sample size, in which, for most participants, the assessment of the medications used was performed at more than one moment (prenatal and perinatal period). To date, this is one of the few studies on this topic carried out in a middle-income country. Several adjustments were also made for known risk factors for PPD.

Our study demonstrated that the apparently existing protective effect between the use of folic acid and DPP disappears after controlling for confounding factors, and several forms of analysis led to the same result. Given that PPD is considered a problem with multifactorial etiology ^37 , 38^ , with negative effects on the woman, the family, and the child, it is important that future studies seek to measure the nutritional status of folate through objective methods, such as serum level measurement.
