# Supplementary material for: Folic acid supplementation during pregnancy and postpartum depressive symptoms
Source: Rev Saude Publica. 2023 Oct 24;57:76. doi: 10.11606/s1518-8787.2023057004962 (PMC10609648; doi:10.11606/s1518-8787.2023057004962)
Supplement: Translation

# Translation: Suplementação de ácido fólico na gestação e sintomas depressivos pós-parto

**Authors:** Bárbara Heather Lutz, Iná da Silva dos Santos, Marlos Rodrigues Domingues, Joseph Murray, Mariângela Freitas da Silveira, Vanessa Irribarem Avena Miranda, Marysabel Pinto Telis Silveira, Sotero Serrate Mengue, Tatiane da Silva dal Pizzol, Andréa Dâmaso Bertoldi

**Affiliations:** I Brasil Universidade Federal de Pelotas. Departamento de Medicina Social. Programa de Pós-Graduação em Epidemiologia. Pelotas, RS, Brasil; II Brasil Universidade Federal de Pelotas. Programa de Pós-Graduação em Educação Física. Pelotas, RS, Brasil; III Brasil Universidade do Extremo Sul Catarinense. Programa de Pós-graduação em Saúde Coletiva. Criciúma, SC, Brasil .; IV Brasil Universidade Federal de Pelotas. Departamento de Fisiologia e Farmacologia. Programa de Pós-Graduação Multicêntrico em Ciências Fisiológicas. Pelotas, RS, Brasil; V Brasil Universidade Federal do Rio Grande do Sul. Faculdade de Medicina. Programa de Pós-Graduação em Epidemiologia. Porto Alegre, RS, Brasil

**Keywords:** Gravidez, Ácido Fólico, Depressão Pós-Parto, Estudos de Coortes

## Abstract

**OBJETIVO:**

Verificar se a suplementação de ácido fólico durante a gestação está associada com a ocorrência de sintomas depressivos maternos aos três meses pós-parto, na Coorte de Nascimentos de Pelotas de 2015.

**MÉTODOS:**

Este estudo incluiu 4.046 mulheres, que foram classificadas em três grupos: sem suplementação de ácido fólico na gestação; uso durante apenas um trimestre da gestação;e uso durante dois ou três trimestres. Os sintomas depressivos foram avaliados aos três meses pós-parto, através da Escala de Depressão Pós-Natal de Edimburgo (EPDS), nos pontos de corte ≥ 10 (sintomas leves) e ≥ 13 (intensidade moderada a grave).

**RESULTADOS:**

A prevalência geral de sintomas leves foi de 20,2% (IC95% 19,0–21,5),e moderados e graves de 11% (IC95% 10,0–12,0). Entre as mulheres que não fizeram uso de ácido fólico, a prevalência de EPDS ≥ 10 foi de 26,8% (IC95% 24,0–29,5) e 18,1% tanto entre as que utilizaram durante um trimestre da gestação (IC95% 16,1–20,1), quanto entre as que utilizaram por dois ou três trimestres (IC95% 16,0–20,2). Já a prevalência de EPDS ≥ 13 foi 15,7% (IC95% 13,5–17,9) entre as que não utilizaram ácido fólico, 9,1% (IC95% 7,5–10,6) entre as que utilizaram durante um trimestre e 9,4% (IC95% 7,8–11,0) entre as que utilizaram por dois ou três trimestres. Nas análises ajustadas, não houve associação estatisticamente significativa entre o uso de ácido fólico na gestação e a ocorrência de sintomas depressivos aos três meses pós-parto.

**CONCLUSÃO:**

Não se observou associação entre a suplementação de ácido fólico na gestação e depressão pós-parto aos três meses.

## INTRODUÇÃO

Os transtornos depressivos são classificados pela Organização Mundial da Saúde (OMS) como o maior contribuinte para a incapacidade global, particularmente entre as mulheres^1 , 2^. São caracterizados por tristeza, perda de interesse ou prazer, sentimento de culpa ou baixa autoestima, alterações no sono ou apetite, sensação de cansaço e falta de concentração, podendo ser duradouros ou recorrentes, prejudicando substancialmente a capacidade do indivíduo de lidar com a vida diária^1 , 2^.

A depressão pós-parto (DPP) é uma complicação comum da gravidez^3^, cuja gravidade depende de fatores como o contexto socioeconômico e o apoio familiar^4^. A DPP traz consequências adversas não apenas para a mãe, mas também para a família, pois pode afetar o desenvolvimento infantil (por exemplo, está associada com maior risco de baixo peso no primeiro ano de vida^5^), pode gerar dificuldade de estabelecimento do vínculo com o bebê^6^e prejuízo nos aspectos sociais, afetivos e no desenvolvimento cognitivo^7 , 8^.

Revisão sistemática com 16 estudos internacionais, incluindo cerca de 35.000 mulheres, indicou prevalência média de 17% de puérperas com alta probabilidade de apresentar DPP. A maioria dos estudos utilizou a Escala de Depressão Pós-Natal de Edimburgo ( *Edinburgh Postnatal Depression Scale* – EPDS), com pontos de corte variando de 9 a 13^9^. Dados de um estudo brasileiro com cerca de 23.000 mulheres, de todas as regiões do país, indicaram prevalência ainda mais elevada, de cerca de 25%, utilizando EPDS ≥ 13^10^.

Acredita-se que a etiologia da DPP esteja ligada a fatores biológicos, genéticos, hormonais, psicossociais e ambientais^3^. A deficiência de fatores nutricionais, como folato/ácido fólico, vitamina B12, ácidos graxos poli-insaturados, selênio, zinco, cálcio e ferro também tem sido associada à DPP^3 , 11^. O ácido fólico faz parte do complexo vitamínico B (vitamina B9). Uma metanálise de estudos observacionais mostrou que baixos níveis de folato estão associados à depressão na população geral^14^. O folato é um dos principais determinantes do metabolismo do carbono-1, no qual a S-Adenosilmetionina (SAM) é formada. A SAM doa grupos metil importantes para a função neurológica. Além disso, o aumento da homocisteína plasmática é um marcador funcional da deficiência de folato, e níveis elevados de homocisteína são encontrados em pacientes depressivos^15 , 16^.

A OMS preconiza a suplementação diária oral de ferro e ácido fólico como parte da assistência pré-natal para reduzir o risco de baixo peso ao nascer, anemia materna e deficiência de ferro^17^. No Brasil, a suplementação com ácido fólico é indicada pelo menos 30 dias antes da concepção até o final da gestação, na dose de 0,4mg por dia, para prevenção de defeitos do tubo neural e anemia^18^. Há poucos estudos publicados que avaliaram a associação entre suplementação de ácido fólico ou os níveis de folato durante a gestação e a ocorrência de DPP^3 , 19^, sendo apenas dois de base populacional^20 , 21^. Assim, o objetivo deste estudo é avaliar a prevalência de sintomas depressivos aos três meses após o parto e sua relação com o uso de suplementação de ácido fólico entre as mães pertencentes à Coorte de Nascimentos de Pelotas de 2015 (C2015).

## MÉTODOS

Os dados deste estudo fazem parte da C2015, realizada na cidade de Pelotas (RS), no sul do Brasil. Todas as mulheres que deram à luz nas cinco maternidades de Pelotas, de 1 de janeiro de 2015 a 31 de dezembro de 2015, e que residiam na zona urbana do município e na colônia Z3, bem como no bairro Jardim América, contíguo a Pelotas e pertencente ao município do Capão do Leão, foram convidadas a participar do estudo. Detalhes metodológicos podem ser encontrados em outra publicação^23^.

Esta coorte iniciou em 2014, no período pré-natal. As 123 unidades de saúde e clínicas privadas que oferecem atendimento pré-natal na cidade foram visitadas semanalmente, entre maio de 2014 e dezembro de 2015, para identificar gestantes com data provável do parto para o ano de 2015. Estas mulheres foram visitadas em casa ou convidadas a comparecer à clínica de pesquisa, entre 16 e 24 semanas de gestação, para responderem a um questionário de saúde, incluindo questões sobre o uso de medicamentos e vitaminas^23^.

No estudo perinatal, as mães foram entrevistadas após o parto, durante sua estadia na maternidade, respondendo um questionário padronizado sobre o período pré-natal, incluindo o uso de medicamentos e vitaminas. Dado que 75% das puérperas haviam sido também entrevistadas durante o estudo pré-natal da C2015, foi possível complementar a informação sobre o uso de medicamentos e vitaminas captada no estudo perinatal com dados obtidos em um momento mais próximo à sua utilização. Essa estratégia permitiu qualificar a informação, tendo em vista que, durante o pré-natal, o período recordatório era menor, e, como as entrevistas foram realizadas nos domicílios, foi possível verificar as prescrições e embalagens dos produtos utilizados.

Tanto no estudo pré-natal quanto no perinatal, as informações referentes ao uso de ácido fólico foram obtidas por meio das seguintes perguntas: “A Sra. usou ou está usando alguma vitamina, cálcio, ácido fólico ou sais de ferro desde que ficou grávida?”. Se sim, os nomes destes medicamentos eram questionados e, posteriormente, para cada medicamento relatado eram realizadas as seguintes perguntas, com o intuito de caracterizar seu uso: “Em que trimestre da gravidez a Sra. usou esse remédio?” 1º trimestre (até 13ª semana), 2º trimestre (entre a 14ª a 27ª semana) e 3º trimestre (28ª semana em diante ou ainda em uso). Para o atual estudo, foi considerado o uso de ácido fólico isolado ou em associação com outras vitaminas e/ou sais minerais, independente da dosagem.

O acompanhamento de três meses após o parto foi realizado na clínica de pesquisa. Nessa ocasião, os sintomas depressivos maternos foram avaliados por meio da EPDS. Esta escala é composta por 10 itens, cada um com pontuação que varia de zero a três. A EPDS foi validada para uso no Brasil, sendo considerado o ponto de corte ≥ 10 como o melhor para identificar mães com sintomas depressivos leves no período pós-parto, com sensibilidade de 82,7% (75,3–89,9) e especificidade de 65,4% (59,8–71,1)^24^; e o ponto de corte ≥ 13 para sintomas depressivos de moderada a grave intensidade, com sensibilidade de 59,6% (49,5–69,1) e especificidade de 88,3% (83,9–91,9)^24^. Para este estudo, foram excluídas as mães que compareceram ao acompanhamento de três meses, mas que não responderam à EPDS.

Os potenciais confundidores analisados foram: idade (< 20, 20–34 e ≥ 35 anos); cor (branca, preta e parda/outra); escolaridade (0–4, 5–8, 9–11 e ≥ 12 anos de estudo); renda em salários-mínimos (SM), considerando o valor de R$ 788,00 em vigor durante o ano de 2015 (≤ 1, 1,1–3, 3,1–6, 6,1–10 e > 10); paridade (1, 2, 3 e ≥ 4 filhos); viver com companheiro (sim/não); apoio do pai do bebê durante a gravidez (muito, mais ou menos/pouco, nenhum apoio); sintomas depressivos na gestação (EPDS ≥ 11)^25^, aplicado no estudo pré-natal; trimestre de início do pré-natal (1º, 2º ou 3º); número de consultas de pré-natal (< 6 ou ≥ 6); tabagismo na gestação (sim/não); uso de álcool na gestação (sim/não); e atividade física na gestação (150 minutos ou mais por semana em pelo menos um trimestre) (sim/não). A idade foi coletada em anos completos e posteriormente categorizada. A cor foi autorreferida pelas mães. A escolaridade foi coletada em anos completos de estudo e, posteriormente, categorizada. A renda familiar foi relatada em reais e posteriormente categorizada em SM. Para a paridade, foi considerada também a gestação atual, sem incluir os natimortos, sendo categorizada posteriormente. Para uma análise complementar, foi considerado o uso de medicamentos antidepressivos do nascimento aos três meses pós-parto (sim/não).

A análise dos dados foi realizada no programa estatístico Stata^®^, versão 12.1. A amostra foi descrita apresentando-se as proporções das variáveis independentes e intervalos de confiança de 95% (IC95%). As mulheres foram classificadas em três grupos em relação à exposição ao ácido fólico: sem uso de ácido fólico na gestação, uso durante apenas um trimestre da gestação e uso de ácido fólico durante dois ou três trimestres. Foi calculada a média e desvio padrão (DP) do escore contínuo da EPDS e a prevalência dos desfechos EPDS ≥ 10 e EPDS ≥ 13, com IC95%, para cada categoria de uso de ácido fólico.

As análises ajustadas foram conduzidas por meio de regressão de Poisson para os dois desfechos: EPDS ≥ 10 e EPDS ≥ 13. As variáveis foram selecionadas em *backward* , sendo mantidas no modelo aquelas com valor p < 0,20. O nível de significância adotado para se considerar as associações estatisticamente significativas foi 0,05. As variáveis idade, escolaridade (anos de estudo), renda, número de consultas de pré-natal e paridade foram analisadas de forma contínua. Todas as variáveis incluídas no modelo foram testadas quanto à sua associação com o uso de ácido fólico e sintomas depressivos pós-parto, apresentando relações estatisticamente significativas.

Adicionalmente, foram realizadas duas análises complementares: A) por regressão logística multinomial, com o desfecho em três categorias: EPDS < 10, EPDS 10 a 12 e EPDS ≥ 13; e B) por regressão linear, com o desfecho EPDS na forma contínua. Foi também realizado teste de interação entre o uso de ácido fólico e sintomas depressivos na gestação (EPDS ≥ 11).

O estudo foi aprovado pelo Comitê de Ética da Escola Superior de Educação Física da Universidade Federal de Pelotas, sob o Protocolo 522.064, registrado na Plataforma Brasil. Todas as entrevistas foram realizadas após a assinatura do termo de consentimento livre e esclarecido pelas mães.

## RESULTADOS

A Coorte de Nascimentos de Pelotas de 2015 é composta pelos nascidos vivos de 4.220 mulheres. Houve 160 perdas no acompanhamento de três meses, e 14 mulheres não responderam ao EPDS. O escore da EPDS aos três meses pós-parto variou de 0 a 27, com a média de 5,99 pontos (DP = 4,83). A prevalência de sintomas depressivos leves (EPDS ≥ 10) foi de 20,2% (IC95% 19,0–21,5), e de sintomas moderados e graves (EPDS ≥ 13), 11,0% (IC95% 10,0–12,0).

Na Tabela 1 estão apresentadas as características da amostra analisada, de acordo com as variáveis independentes e a comparação com a coorte original (amostra inicial coletada no estudo perinatal). A maioria das puérperas referiu ser branca (70,8%), idade entre 20 e 34 anos (70,8%), viver com companheiro (85,8%), ter recebido muito apoio do pai do bebê durante a gravidez (89%), ter iniciado o acompanhamento pré-natal no primeiro trimestre gestacional (55,5%) e haver realizado seis ou mais consultas (86,6%). Pouco mais de um terço (34,5%) tinha 9 a 11 anos de estudo, cerca da metade (47,2%) tinha renda familiar de 1,1 a 3 SM e 50,0% eram primíparas. Com relação a comportamentos de saúde, 7,4% relataram ter consumido álcool no período gestacional, 16,1% eram tabagistas, e apenas 10,1% praticaram atividade física neste período. Entre as mulheres que haviam sido captadas no estudo pré-natal (n = 3.029), 24% apresentaram EPDS ≥ 11. Não houve diferenças estatisticamente significativas entre a coorte original e a coorte analisada em relação às variáveis independentes ( Tabela 1 ).

**Tabela 1:** Comparação entre a coorte original e as participantes do estudo. Coorte de Nascimentos de Pelotas, 2015.

Características maternas	Coorte original (n = 4.220)		Participantes* (n = 4.046)	
	
	n	% (IC95%)	n	% (IC95%)
Idade (anos)				
< 20	619	14,7 (13,6–15,7)	593	14,7 (13,6–15,8)
20–34	2.981	70,7 (69,3–72,0)	2.864	70,8 (69,4–72,2)
≥ 35	619	14,7 (13,6–15,7)	588	14,5 (13,4–15,6)
Renda familiar (salários-mínimos)				
≤ 1	534	12,7 (11,7–13,7)	506	12,5 (11,5–13,5)
1,1–3,0	1.991	47,2(45,7–48,7)	1.910	47,2 (45,7–48,8)
3,1–6,0	1.115	26,4 (25,1–27,8)	1.077	26,6 (25,3–28,0)
6,1–10,0	316	7,5 (6,7–8,3)	299	7,4 (6,6–8,2)
> 10,0	262	6,2 (5,5–6,9)	252	6,2 (5,5–7,0)
Escolaridade (anos de estudo)				
0–4	387	9,2 (8,3–10,0)	361	8,9 (8,0–9,8)
5–8	1.084	25,7 (24,4–27,0)	1.041	25,7 (24,2–27,1)
9–11	1.442	34,2 (32,7–35,6)	1.397	34,5 (33,1–36,0)
≥ 12	1.306	31,0 (29,6–32,4)	1.246	30,8 (29,4–32,2)
Cor				
Branca	2.982	70,8 (69,4–72,2)	2.861	70,8 (69,4–72,2)
Preta	661	15,7 (14,6–16,8)	635	15,7 (14,6–16,8)
Parda/outra	570	13,5 (12,5–14,6)	543	13,4 (12,4–14,5)
Vive com companheiro				
Sim	3.620	85,8 (84,7–86,9)	3.471	85,8 (84,7–86,9)
Não	599	14,2 (13,1–15,3)	574	14,2 (13,1–15,3)
Apoio do pai do bebê				
Muito apoio	3.690	89,0 (88,1–90,0)	354	89,0 (88,0–90,0)
Mais ou menos/pouco apoio	297	7,2 (6,4–8,0)	288	7,2 (6,4–8,0)
Nenhum apoio	157	3,8 (3,2–4,4)	150	3,8 (3,2–4,4)
Paridade (apenas nascidos vivos)				
1	2.108	50,0 (48,5–51,5)	2.023	50,0 (48,5–51,6)
2	1.306	31,0 (29,6–32,4)	1.260	31,1 (29,7–32,6)
3	4.461	10,9 (10,0–11,9)	443	11,0 (10,0–11,9)
≥ 4	343	8,1 (7,3–9,0)	319	7,9 (7,1–8,7)
Número consultas de pré-natal				
< 6	577	14,0 (13,0–15,1)	531	13,4 (12,3–14,5)
≥ 6	3.538	86,0 (84,9–87,0)	3.429	86,6 (85,5–87,7)
Trimestre de início do pré-natal				
Primeiro	2.058	54,9 (53,3–56,5)	2.013	55,5 (53,8–57,1)
Segundo	1.461	39,0 (37,4–40,6)	1.396	38,5 (36,9–40,0)
Terceiro	228	6,1 (5,3–6,9)	221	6,1 (5,3–6,9)
Sintomas depressivos na gestação**				
Sim	746	24,2 (22,6–25,7)	726	24,0 (22,4–25,5)
Não	2.342	75,8 (74,3–77,4)	2.303	76,0 (74,5–77,6)
Uso de álcool na gestação				
Sim	314	7,4 (6,7–8,2)	301	7,4 (6,6–8,3)
Não	3.903	92,6 (91,8–93,3)	3.743	92,6 (91,7–93,4)
Tabagismo na gestação				
Sim	698	16,6 (15,4–17,7)	653	16,1 (15,0–17,3)
Não	3.519	83,4 (82,3–84,6)	3.391	83,9 (8,27–85,0)
Atividade física na gestação (≥ 150 min/semana em pelo menos um trimestre)				
Sim	423	10,0 (9,1–10,9)	407	10,1 (9,1–11,0)
Não	3.797	90,0 (89,1–90,9)	3.639	89,9 (89,0–90,9)
Uso de ácido fólico na gestação				
Sim	3.080	74,1 (72,8–75,4)	2.966	74,4 (73,0–75,7)
Não	1.076	25,9 (24,6–27,2)	1.021	25,6 (24,3–27,0)

IC95%: intervalo de confiança de 95%.

*Participantes do acompanhamento de 3 meses com informação sobre sintomas depressivos pós-parto.

**n = 3.088 mulheres avaliadas no estudo pré-natal através da escala de Edimburgo, considerando o ponto de corte ≥ 11^25^.

Entre as 4.046 mulheres incluídas na atual análise, 3.987 relataram informação sobre o uso de ácido fólico. Dentre estas, 2.966 (74,4%; IC95% 73,0–75,7) informaram ter usado ácido fólico durante a gestação. Entre as que usaram, 88,4% (n = 2,621) fizeram uso, em algum momento da gestação, de suplemento exclusivo de ácido fólico (sem associações com outras vitaminas ou sais minerais). A Figura mostra os trimestres de uso de ácido fólico pelas mulheres participantes do estudo. Das 2.966 mulheres que relataram ter usado ácido fólico durante a gestação, apenas 2.689 informaram o trimestre de uso. Dentre estas, 43,8% utilizaram ácido fólico apenas durante o primeiro trimestre.

**Figura 1:** Trimestres de uso de ácido fólico na gestação (n = 2.689). Coorte de Nascimentos de Pelotas, 2015.

A Tabela 2 mostra a média e DP do EPDS aos três meses e a prevalência dos desfechos EPDS ≥ 10 e ≥ 13 de acordo com as categorias de exposição. Entre as mulheres que não utilizaram ácido fólico na gravidez, a média da EPDS foi de 6,8 pontos (DP = 5,3); entre as que utilizaram durante um trimestre, de 5,7 (DP = 4,7); e entre as que utilizaram por dois ou três trimestres, de 5,7 (DP = 4,6).

**Tabela 2:** Média e desvio-padrão do escore contínuo e prevalências dos desfechos em cada categoria de exposição. Coorte de Nascimentos de Pelotas, 2015.

Exposição	Média e DP escore EPDS	EPDS ≥ 10	EPDS ≥ 13
	
		(IC95%)	(IC95%)
Sem uso de ácido fólico (n = 1.020)	6,8 (5,3)	26,8 (24,0–29,5)	15,7 (13,5–17,9)
Uso em 1 trimestre (n = 1.391)	5,7 (4,7)	18,1 (16,1–20,1)	9,1 (7,5–10,6)
Uso em 2 ou 3 trimestres (n = 1.298)	5,7 (4,6)	18,1 (16,0–20,2)	9,4 (7,8–11,0)

DP: desvio-padrão; EPDS: Escala de Depressão Pós-natal de Edimburgo; IC95%: intervalo de confiança de 95%.

Entre as mulheres que não fizeram uso de ácido fólico, a prevalência de EPDS ≥ 10 foi 26,8% (IC95% 24,0–29,5), e de 18,1% tanto entre as que utilizaram durante um trimestre da gestação (IC95% 16,1–20,1) quanto entre as que utilizaram por dois ou três trimestres (IC95% 16,0–20,2) ( Tabela 2 ). Já a prevalência de EPDS ≥ 13 foi de 15,7% (IC95% 13,5–17,9) entre as mulheres que não utilizaram ácido fólico, 9,1% (IC95% 7,5–10,6) entre as que utilizaram durante um trimestre e 9,4% (IC95% 7,8–11,0) entre as que utilizaram por dois ou três trimestres ( Tabela 2 ).

A Tabela 3 mostra as razões de prevalência para EPDS ≥ 10 e EPDS ≥ 13, com os respectivos IC95%, obtidos na análise bruta e nos modelos ajustados. Para sintomas depressivos leves (EPDS ≥ 10), as variáveis que permaneceram para ajuste no modelo final foram escolaridade, apoio do pai do bebê, uso de álcool na gestação, paridade e sintomas depressivos na gestação. Já para sintomas moderados e graves (EPDS ≥ 13), as variáveis que permaneceram no modelo final foram “viver com companheiro”, “paridade” e “sintomas depressivos na gestação”.

**Tabela 3:** Associações entre uso de ácido fólico na gestação e sintomas depressivos pós-parto. Coorte de Nascimentos de Pelotas, 2015.

Variáveis	Análise bruta (n = 3.709)		Análise ajustada (n = 2.810)*	
	
	RP (IC95%)	Valor p	RP (IC95%)	Valor p
EPDS ≥ 10				
Sem uso de ácido fólico	1	< 0,001	1	0,112
Uso em um único trimestre	0,68 (0,58–0,79)		0,96 (0,80–1,14)	
Uso em dois ou três trimestres	0,68 (0,58–0,79)		0,87 (0,72–1,04)	
EPDS ≥ 13				
Sem uso de ácido fólico	1	< 0,001	1	0,107
Uso em um único trimestre	0,58 (0,46–0,72)		0,84 (0,65–1,10)	
Uso em dois ou três trimestres	0,60 (0,48–0,75)		0,80 (0,61–1,04)	

IC95%: intervalo de confianca de 95%; EPDS: Escala de Depressao Pos-Natal de Edimburgo.

*Análise ajustada (Regressão de Poisson) para idade, escolaridade, cor, renda, paridade, viver com companheiro, apoio do pai do bebê, trimestre de início do pré-natal, número de consultas pré-natal, tabagismo na gestação, uso de álcool na gestação, atividade física na gestação e sintomas depressivos na gestação (EPDS ≥ 11).

Na análise bruta, o uso de ácido fólico apresentou associação estatisticamente significativa com diminuição do risco de sintomas depressivos pós-parto aos três meses, tanto entre as usuárias de ácido fólico por apenas um trimestre como entre aquelas que usaram por dois trimestres ou mais, em ambos os pontos de corte da EPDS ( Tabela 3 ). Já nas análises ajustadas, não houve associação estatisticamente significativa entre o uso de ácido fólico na gestação e a ocorrência de sintomas depressivos aos três meses pós-parto, considerando os dois pontos de corte.

Nas análises complementares, tanto na regressão logística multinomial quanto na regressão linear, os resultados foram semelhantes e no mesmo sentido das análises por regressão de Poisson. O teste de interação não indicou que a variável “sintomas depressivos na gestação” modificasse a associação entre uso de ácido fólico e sintomas depressivos no período pós-parto. Adicionalmente, também foi realizado controle para o uso de antidepressivos, não havendo modificações nos resultados. Apenas 23 mulheres estavam em uso de antidepressivos do nascimento aos três meses pós-parto, correspondendo a 0,5% da amostra. Sete delas apresentaram pontuação acima de 10 no questionário Edimburgo, sendo quatro delas com sintomas moderados a graves.

## DISCUSSÃO

A prevalência de sintomas depressivos leves aos três meses pós-parto foi de 20,2% e de sintomas depressivos de moderada a grave intensidade, 11%. O uso de ácido fólico durante a gestação mostrou efeito protetor na análise bruta para ambos os pontos de corte, porém perdeu significância nas análises ajustadas.

Diversos estudos relataram a relação entre folato e depressão na população geral^14 , 16 , 26 , 27^, mas não há muitas evidências em relação à DPP. Um estudo observou baixos níveis séricos de folato e vitamina B12 e níveis elevados de homocisteína em mulheres em idade fértil com transtornos psicóticos^28^. Os suplementos de ácido fólico já foram estudados como tratamento adjuvante da depressão^15 , 29^, e dietas ricas em ácido fólico sugeriram redução no risco de depressão em algumas populações^30 , 31^. O estudo de Yan^3^encontrou um risco menor para DPP em gestantes que haviam feito suplementação de ácido fólico por mais de seis meses, em comparação com aquelas que suplementaram por menos de seis meses. Possíveis hipóteses para essa diferença e limitações desse estudo seriam a exclusão de mulheres que não usaram ácido fólico ou que utilizaram só no período pré-concepcional e a não inclusão de sintomas de depressão durante o período gestacional como fator confundidor. O estudo foi realizado com mulheres que fizeram revisão pós-parto em centros de saúde materno-infantil em Tianjin, China, e utilizou uma escala designada para o *screening* de sintomas depressivos na população em geral^3^.

Outros estudos não encontraram relação entre suplementação de ácido fólico/níveis de folato e depressão pós-parto. Blunden et al.^21^não encontraram diferenças significativas entre mulheres com ou sem sintomas depressivos pós-parto em relação à concentração de folato nos glóbulos vermelhos ou ingestão alimentar de folato, vitamina B12 e vitamina B6, antes ou durante a gravidez. Chong et al.^19^também não observaram diferenças nas concentrações plasmáticas de folato em mulheres com e sem DPP, porém as concentrações de folato foram significativamente mais baixas entre aquelas com provável depressão gestacional do que naquelas sem sintomas. Miyake et al.^22^não observaram associação entre a ingestão de folato, cobalamina ou piridoxina e o risco de DPP. No estudo de Lewis et al.^20^não foram encontradas evidências de que a suplementação de ácido fólico reduziria o risco de depressão durante a gravidez e até oito meses pós parto. Entretanto, o mesmo estudo mostrou que os suplementos de ácido fólico durante a gravidez protegeram contra depressão 21 meses após o parto, e que esse efeito foi mais pronunciado entre as mulheres com o genótipo MTHFR C677T TT.

A plausibilidade para o potencial efeito protetor do folato sobre a ocorrência de DPP decorre do fato de que esse e outros nutrientes são importantes no sistema de neurotransmissão, e a gravidez tende a levar a sua depleção^12 , 13 , 32^. As concentrações de folato no soro materno e nos eritrócitos diminuem a partir do quinto mês de gravidez e costumam permanecer baixas por um longo período após o parto^33^. Folato, vitamina B12 e vitamina B6 são fatores críticos no metabolismo da homocisteína, que é um precursor necessário na biossíntese dos neurotransmissores serotonina, dopamina e norepinefrina, que estão implicados na patogênese da depressão^27 , 32^.

Dois ensaios randomizados, publicados no início da década de 1990, demonstraram que a suplementação de ácido fólico evitava a ocorrência e a recorrência de defeitos do tubo neural^34 , 35^, sendo então recomendada a suplementação universal no período pré-concepcional e durante a gestação para a prevenção destes defeitos, bem como para prevenção de anemia^17 , 18^. No período do estudo, o protocolo vigente no Brasil recomendava o uso de ácido fólico durante o período pré-concepcional e apenas no primeiro trimestre gestacional^4^. Porém, é comum não haver a prescrição deste suplemento quando a mulher começa o pré-natal tardiamente^36^.

Esse estudo apresenta algumas limitações. Não foi possível avaliar a dosagem dos suplementos utilizados, tendo sido analisada apenas a informação de utilização por trimestre gestacional, porém sem a garantia de que o suplemento tenha sido utilizado durante todo o período do trimestre em questão. Além disso, nossa análise baseia-se no autorrelato, não tendo sido feito recordatório da dieta, do consumo de alimentos fortificados com ácido fólico ou da dosagem de folato sérico. Da mesma forma, não foi possível avaliar o uso de suplementação no período pré-concepcional.

Entre os pontos fortes deste estudo encontram-se o fato de que é uma coorte de base populacional, com grande tamanho de amostra, em que, para a maioria das participantes, a avaliação dos medicamentos utilizados foi realizada em mais de um momento (período pré-natal e perinatal). Até o momento, este é um dos poucos estudos sobre essa temática realizados em um país de renda média. Também foram realizados diversos ajustes para conhecidos fatores de risco para DPP.

Nosso estudo demonstrou que o efeito de proteção aparentemente existente entre o uso de ácido fólico e DPP desparece após controle para fatores de confusão, sendo que várias formas de análise levaram ao mesmo resultado. Tendo em vista que a DPP é considerada um problema de etiologia multifatorial^37 , 38^, com efeitos negativos sobre a mulher, a família e a criança, é importante que futuros estudos busquem mensurar o status nutricional de folato por meio de métodos objetivos, como a dosagem sérica.
